# Account of Some Cases of the Plague, Which Occurred on Board of a British Ship of War, on the Coast of Syria

**Published:** 1801-06

**Authors:** 


### *Account of some Cases of the Plague, which occurred on board of a British Ship of War, on the Coast of Syria. Communicated by Dr.* Blane.

In the ſpring and ſummer of 1799, the plague prevailed in Syria, and was communicated to ſome of the Britiſh ſhips of war employed on that coaſt, particularly at the celebrated ſiege of St. Jean D'Acre. An account of ſome of theſe caſes was ſent by Mr. Tainſh, ſurgeon of the Theſeus, a ſeventy-four gun ſhip, to the Commiſſioners of ſick and wounded Seamen; and as there ſeems to be no doubt that theſe were caſes of the true plague, from the deſcription, as well as from the exiſtence of it on the ſpot at that time, as opportunities of obſerving it rarely occur to practitioners of this country, and as it was ſucceſsfully treated by powerful medicines, theſe caſes ſeem deſerving of being communicated to the public, particularly at this moment, when the defenders of our country are expoſed to that dreadful malady in Egypt.

The greater part of the ſymptoms enumerated are ſuch as might occur in a violent fever; but the buboes which were obſerved in the axilla in three caſes, and in the groin in two caſes, out of the five which occurred, ſerved, in conjunction with the other ſymptoms, to characterize and aſcertain the diſeaſe. The moſt remarkable ſymptoms beſides theſe were violent vomiting, *petechiæ*, ſwelled tongue, redneſs of the eyes, ſevere head-ach, and great debility; rigors, foul tongue, and the other concomitants of fever alſo attended it. But it will be more ſatisfactory to give the moſt intereſting and inſtructive parts of Mr. Tainſh's narrative in his own words.

" The firſt caſe was that of Colonel Philipeaux, a French officer, acting under the orders of Sir Sydney Smith, at the ſiege of Acre. He was always fearful of the plague, and dreaded much the touch of any Turk, which kept his mind in a ſtate of continual anxiety. When ſeized with the diſeaſe he would take no medical advice, but drank copiouſly of lemonade. He died the fourth day of his illneſs by a hæmorrhage from the bowels, with other ſymptoms of a moſt ſevere fever. His bed and bedding were thrown overboard with him, and every precaution was taken to fumigate and cleanſe the captain's cabin where he died.

" No other caſe occurred till we took ſeveral French boats off Jaffa, with wounded and ſick men, after their departure from the ſiege of Acre. Thoſe apparently in health, particularly the ſeamen, were ordered on board the Theſeus, and one of of them was ſeized with the ſymptoms above deſcribed, which in twenty-four hours were followed by buboes and extreme debility. Having at that time no ſick, I removed him to the ſick birth, ordered every part of his clothes off, and himſelf to be waſhed from head to foot with ſoap and warm water, after which his head, arm-pits, and pubis were ſhaved. He was furniſhed with a clean bed, linen, and night-cap, and all his former clothing was thrown overboard. I then gave him an emetic, conſiſting of half a drachm of ipecacuanha, and a grain and a half of tartar emetic; which not having operated freely, it was repeated in three hours, and he diſcharged an enormous quantity of bile, viſcid fordes, and tough phlegm. I next gave him ten grains of calomel and ſix grains of antimonial powder, and repeated them in four hours; he had, at the ſame time, a laxative clyſter, which procured him ſeveral ſtools that ſeemed to give him much relief. He reſted pretty well, and having ſome fever next day the antimonial was continued. Towards the evening he had an exacerbation, attended with delirium, which left him after the application of a bliſter to the head. He now complained of a bitter taſte and nauſea, with great proſtration of ſtrength. I gave him a ſcruple of ipecacuanha and two grains of tartar emetic, which cleared the ſtomach of a large quantity of diſagreeable matter, which gave him great eaſe. As he was now free from fever, I determined to give him the Peruvian bark, which was mixed with Port wine, and he took it with much pleaſure, making ſigns for more. In an hour I let him drink half a pint of Port alone, which pleaſed him much; and after this he ſlept above three hours. On waking he was much refreſhed. At bed-time I gave him ſix grains of camphor and two of opium, which procured him a good night, and next day he was greatly better. As he expreſſed much inclination for wine, I gave him ſome of the red wine with bark infuſed in it; but he preferred the wine by itſelf, which was very good, and by promiſing him plenty I prevailed on him to take the infuſion alternately with it, regulating the quantity according to his ſenſations.

" The buboes continued ſtationary during the early part of the fever; but as ſoon as he began to take wine and nouriſhment, they aſſumed an inflammatory appearance, with excruciating pain. I ordered warm fomentations and emollient poultices, and he took an opiate at bed-time. As theſe poultices did not ſeem to aſſiſt maturation, I ordered others, conſiſting of ſoap, aſſafœtida, and onions chopped ſmall, which had the deſired effect in bringing them to ſuppuration. I opened them with the lancet; the matter was of a thin ſanious nature. The poultice being continued, the glands in a cluſter protruded

Z z z 2 through through the opening, without any fuppuration having taken place in their fubftance. I cut them away, and dreffed the fore with an ointment compofed of yellow bafilicon, mercury and turpentine, applying the emollient cataplafm over all; and, in a few days, a good pus was produced. He gathered ftrength daily, fometimes drinking wine as far as two quarts, feldom lefs than three pints, in a day; he had alfo as good a diet as the ward-room table could afford. The cure was foon compleated, much to my fatisfaction, and he returned fome time after to France in perfect health.

"The third cafe was that of a feaman, who was fent from the Thefeus on board of a gun-boat. Having communication with the French boats which we took, and having drank a large quantity of fpirituous liquor, he was brought on board in a ftate of low delirium, his tongue being fwelled and protruded, his fkin being dry, and his mouth parched; he had alfo a burning fenfation over the whole body, with fwellings in the arm-pits, *petechiæ*, and univerfal debility. I could not get him to fwallow any thing, and he died in twenty-four hours.

"The fourth cafe was that of our fhip's corporal, Matthew Garland, who had been on board a large boat lying along-fide, to diftribute wine and bread to fome fick and wounded Frenchmen; their wounds had not been dreffed for three days; they were dreffed in the boat, and fupplied with more dreffings. This man was feized with giddinefs and reeling, as if drunk, his eyes rolling, and his tongue fwelled, protruded, and foul; he had alfo naufea and retching, violent head-ach, fhivering, and extreme debility. All his clothes and bedding were thrown over board, himfelf wafhed well with foap, warm water, and vinegar, and fhaved, as mentioned in the fecond cafe. An emetic was adminiftered, and repeated three times in the courfe of twelve hours, which evacuated bile with a mixture of phlegm. At night, after the third emetic had operated, I gave him ten grs. of calomel and eight of antimonial powder, and next morning rhubarb with infufion of fenna, tamarinds, and cream of tartar, which produced vomiting and purging, after which fucceeded a favourable change in all the fymptoms, except in the bubo which was fituated in the left groin, attended with great pain. I gave him bark, antimonials, wine, vitriolic æther with opium at bed-time, and treated the bubo as in the fecond cafe. The wine, bark, æther, and opium were continued, particular attention was paid to the ftate of the bowels, and with thefe remedies and a good diet he foon recovered.

"The fifth cafe was that of a Frenchman. After taking emetics, calomel, and rhubarb, which operated freely, he was fent

ſent on ſhore to Alexandria, and I learned afterwards from a French officer that he recovered."

Mr. Tainſh obſerves at the concluſion of his letter, that the only precaution he ever took againſt infection was, previouſly to touching the perſon, to rub his hands ſlightly over with olive oil, and on leaving the ſick-birth, to waſh it off with warm vinegar, water and ſoap.

Though it is to be regretted that there are ſome points of importance, which have been omitted in the narrative of theſe caſes, ſuch as the interval between the time of receiving the infection and the appearance of the diſeaſe; the particular day of the diſeaſe on which certain ſymptoms aroſe, on which certain remedies were employed, and on which death took place; and though the treatment of the local complaint may appear to ſome not agreeable to the rules of correct ſurgery, yet there are other points ſo inſtructive and novel, as to render this communication very intereſting.

Though the caſes are not ſufficiently numerous to be conſidered as the grounds of an eſtabliſhed practice, yet it muſt be confeſſed that the method of cure was undertaken upon very rational principles, and founded on the analogy of the treatment of other diſeaſes. All febrile diſeaſes admit of ſpontaneous recovery; and a patient may, no doubt, occaſionally ſurvive, in ſpite of improper practice; but the means employed in theſe caſes were of ſo active a nature, that they could hardly have failed to have proved viſibly pernicious, had they not been adapted to the complaint. From the terror naturally excited by this diſeaſe, there is a great want of practical facts; and Mr. Tainſh having, much to his honour, attended theſe caſes with great deliberation and conſtancy of mind, his method of treatment ſeems highly deſerving of notice and imitation.

The methods of prevention that were uſed are no leſs deſerving of attention. The minute accuracy and great pains with which all adhering infection was deſtroyed, cannot be too much commended; and it is to be preſumed that the ſafety of the attendants and ſhip's company was owing to this.

Mr. Tainſh obſerves, that this diſeaſe is not ſo contagious as is commonly ſuppoſed, and aſcribes its ariſing and conſtantly prevailing in Turkey, and not in the reſt of Europe, to the uncleanly habits, and to the careleſsneſs, indolence, and indifference of the Mahomedans, in conſequence of the religious tenet of predeſtination. When the plague was in this country, it chiefly affected the loweſt order of the people, who lived in ill-ventilated habitations, and were uncleanly in their perſons. This is a remark of Lord Clarendon, in the Hiſtory of his own Life; and he ſays, that upon his return to London, he and the other other people of condition, hardly miſſed one of their acquaintances who remained there during the plague. It has long been aſcertained that the ſphere of peſtilential contagion is very ſmall; and ſome are of opinion that abſolute contact is neceſſary in order to its being caught. However this may be, it is evident that it peculiarly affects the inhalants of the ſkin, and not the organs and avenues of reſpiration, like moſt other infectious febrile diſeaſes. All the peculiar diſcriminating ſymptoms of the plague are in proof of this, namely, the buboes, parotids, and carbuncles. It is remarked by Dr. Ruſſell, with his uſual accuracy of obſervation, that the glands of the groin which are affected with buboes in the plague, are not thoſe which are affected in the venereal diſeaſe, but thoſe ſituated under them, through which the lymphatics of the lower extremities paſs. The ſwellings of the axillary and parotid glands are alſo in the ſituations we ſhould expect from the abſorption of virus by thoſe parts of the ſurface of the body which are moſt expoſed to the air, and the contact of external bodies. Carbuncles are alſo in proof of the connection of this diſeaſe with cutaneous abſorption; and upon theſe grounds we cannot but allow that the friction with oil had probably a great effect in preventing the abſorption of the peſtilential virus, and that this, as well as the other facts contained in this communication, ought to be made known to thoſe who are likely to be expoſed to this contagion, or whoſe duty it is to give directions for the prevention of this dreadful epidemic.

